# Bioinformatics tools developed to support BioCompute Objects

**DOI:** 10.1093/database/baab008

**Published:** 2021-03-30

**Authors:** Janisha A Patel, Dennis A Dean, Charles Hadley King, Nan Xiao, Soner Koc, Ekaterina Minina, Anton Golikov, Phillip Brooks, Robel Kahsay, Rahi Navelkar, Manisha Ray, Dave Roberson, Chris Armstrong, Raja Mazumder, Jonathon Keeney

**Affiliations:** The Department of Biochemistry & Molecular Medicine, The George Washington University School of Medicine and Health Sciences, Washington, DC 20037, USA; Seven Bridges, Charlestown, MA 02129, USA; The Department of Biochemistry & Molecular Medicine, The George Washington University School of Medicine and Health Sciences, Washington, DC 20037, USA; The McCormick Genomic and Proteomic Center, The George Washington University, Washington, DC 20037, USA; Seven Bridges, Charlestown, MA 02129, USA; Seven Bridges, Charlestown, MA 02129, USA; CBER-HIVE, Center for Biologics Evaluation and Research, US Food and Drug Administration, Silver Spring, MD 20993, USA; CBER-HIVE, Center for Biologics Evaluation and Research, US Food and Drug Administration, Silver Spring, MD 20993, USA; Seven Bridges, Charlestown, MA 02129, USA; The Department of Biochemistry & Molecular Medicine, The George Washington University School of Medicine and Health Sciences, Washington, DC 20037, USA; The Department of Biochemistry & Molecular Medicine, The George Washington University School of Medicine and Health Sciences, Washington, DC 20037, USA; Seven Bridges, Charlestown, MA 02129, USA; Seven Bridges, Charlestown, MA 02129, USA; The Department of Biochemistry & Molecular Medicine, The George Washington University School of Medicine and Health Sciences, Washington, DC 20037, USA; The Department of Biochemistry & Molecular Medicine, The George Washington University School of Medicine and Health Sciences, Washington, DC 20037, USA; The McCormick Genomic and Proteomic Center, The George Washington University, Washington, DC 20037, USA; The Department of Biochemistry & Molecular Medicine, The George Washington University School of Medicine and Health Sciences, Washington, DC 20037, USA

## Abstract

Developments in high-throughput sequencing (HTS) result in an exponential increase in the amount of data generated by sequencing experiments, an increase in the complexity of bioinformatics analysis reporting and an increase in the types of data generated. These increases in volume, diversity and complexity of the data generated and their analysis expose the necessity of a structured and standardized reporting template. BioCompute Objects (BCOs) provide the requisite support for communication of HTS data analysis that includes support for workflow, as well as data, curation, accessibility and reproducibility of communication. BCOs standardize how researchers report provenance and the established verification and validation protocols used in workflows while also being robust enough to convey content integration or curation in knowledge bases. BCOs that encapsulate tools, platforms, datasets and workflows are FAIR (findable, accessible, interoperable and reusable) compliant. Providing operational workflow and data information facilitates interoperability between platforms and incorporation of future dataset within an HTS analysis for use within industrial, academic and regulatory settings. Cloud-based platforms, including High-performance Integrated Virtual Environment (HIVE), Cancer Genomics Cloud (CGC) and Galaxy, support BCO generation for users. Given the 100K+ userbase between these platforms, BioCompute can be leveraged for workflow documentation. In this paper, we report the availability of platform-dependent and platform-independent BCO tools: HIVE BCO App, CGC BCO App, Galaxy BCO API Extension and BCO Portal. Community engagement was utilized to evaluate tool efficacy. We demonstrate that these tools further advance BCO creation from text editing approaches used in earlier releases of the standard. Moreover, we demonstrate that integrating BCO generation within existing analysis platforms greatly streamlines BCO creation while capturing granular workflow details. We also demonstrate that the BCO tools described in the paper provide an approach to solve the long-standing challenge of standardizing workflow descriptions that are both human and machine readable while accommodating manual and automated curation with evidence tagging.

**Database URL:**  https://www.biocomputeobject.org/resources

## Introduction

The availability of high-throughput sequencing (HTS) data, also referred to as next-generation sequencing (NGS) data, is growing at exponential rates due to decreasing costs to generate, store and analyze NGS data. Similarly, bioinformatics in support of NGS analysis are evolving rapidly: every day novel algorithms are published, researchers generate new interpretations and applications for existing workflows and regulatory sponsors submit data and analysis as regulatory evidence for maintenance and review to regulatory bodies. Platforms such as the High-performance Integrated Virtual Environment (HIVE) (1, 2), Cancer Genomics Cloud (CGC) (3) and Galaxy (4) contain robust infrastructure to support this research—from the start of an analysis to summarizing results and to validation checks that ensure workflow reproducibility. Many community efforts and standards have surfaced in attempts to harmonize the genomics field, including data standards (5, 6), standards for transmitting the data (7), standards for workflows (8–11) and standards for packaging resources (12). Despite these efforts, methods for describing bioinformatics pipelines frequently omit important data, making comprehension of various steps or an entire pipeline difficult or impossible. It is clear that there is a strong need for a descriptive standard that includes pipeline metadata, details for appropriate pipeline execution, parameters chosen and background information not otherwise described (13). The IEEE (Institute of Electrical and Electronics Engineers) 2791-2020 standard, also known as BioCompute, aims to address the growing need to communicate and exchange bioinformatics workflows, especially NGS analysis (14).

IEEE 2791-2020 is the culmination of a multi-community effort led by what is now the BioCompute Public Private Partnership. IEEE 2791-2020 supports interoperability between biomedical researchers, pharmaceutical partners, software developers and regulatory agencies such as the US Food and Drug Administration (FDA). An instance of a pipeline documented according to the IEEE standard is called a BioCompute Object (BCO). BCOs adhere to the BioCompute Specification (15, 16) to encode information on a domain-specific knowledge base and computational workflow execution, metadata, data provenance and appropriate usage within a JavaScript Object Notation (JSON) Schema (17). In addition, BCOs include all the necessary information for understanding and identifying resources required to execute the workflow.

The need for a descriptive standard was particularly felt at the FDA. Pipeline descriptions received during regulatory review are typically received in an ad hoc format, which results in substantially delayed review times due to the need to clarify bioinformatics details (personal communication). The FDA’s Genomics Working Group held a session to develop guidelines for a potential standard with the aim of increasing the efficiency by which bioinformatics workflows can be communicated. The result of this meeting formed the basis for what is now known as BioCompute. Once standardized, BioCompute became officially known as IEEE 2791-2020, which has since been accepted for use in four drug applications at three FDA Centers (18).

To facilitate easier adoption of the standard, several tools for documenting existing workflows with the standard have been developed. Here, we offer an initial evaluation of the first generation of publicly available tools developed to produce BCOs, including the HIVE BCO App, CGC BCO App (19, 20), Galaxy BCO API extension and the BCO Portal. The HIVE BCO App, CGC BCO App and Galaxy BCO API extension are platform-specific tools that facilitate quick and easy pipeline export as IEEE 2791-2020 BCOs. The BCO Portal consists of an interface for building and viewing BCOs and a database of existing BCOs. The BCO Portal demonstrates the ease with which a BCO can be built with little knowledge of the standard.

This paper reports the availability of platform-independent and platform-dependent BCO creation tools and the evaluation of these tools through a novel research initiative with novice bioinformaticians as the initial audience for the platform-independent (BCO Portal) and platform-dependent (HIVE BCO App and CGC BCO App) tools. We then report on the efficacy on which novice bioinformaticians can generate BCOs after conducting platform-specific trainings (HIVE and CGC). BCO Tool users applied these trainings to create BCOs from available RNA-Seq workflows using the CGC BCO App and BCO Portal. Users submitted BCOs to the beginner track of the precisionFDA (pFDA) BCO App-a-thon (21), allowing for systematic BCO evaluation. In addition, we present User Stories on how BCOs generated by these new tools are advancing FAIR principles (22) in existing applications. Lastly, we report on recommended standard and tool revisions aimed to address user questions and challenges identified by the user.

## Methods

The following sections outline the design and development of BCO generation tools that support a variety of computational environments and use cases.

### HIVE BCO App

#### Platform

HIVE is an encapsulated data platform which contains an archival system and compute system integrated behind a common firewall. See Supplementary Materials Section I for more information on HIVE (1, 2).

#### Tool

**Figure 1.**
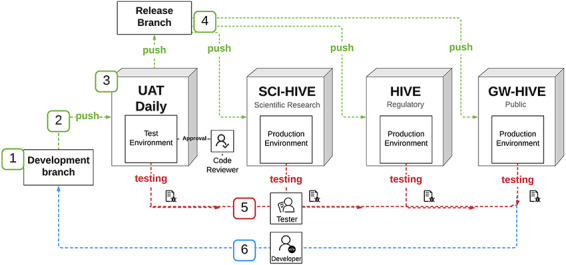
HIVE BCO App deployment diagram.

HIVE architecture provides a backbone for the modular addition of tools. Within this framework, the functionality to record a workflow as a BCO was integrated to record a workflow as a BCO into the HIVE platform by referencing the BioCompute Specification Document. Sections of the specification document were partitioned into those deemed most logical for end users. This planning phase resulted in three broad categories into which the specification could be divided: Provenance Domain, Pipeline Computations and Extra Information. The Provenance Domain tab contains user populated information pertinent to the validity of the BCO. In the second section, Pipeline Computations, objects used for the pipeline are received through user input: comma separated object IDs or directly from the URL. See Supplementary Materials Section II for further details on HIVE BCO App tool implementation. The HIVE BCO App deployment diagram can be found in Figure 1.

Once the (1) HIVE BCO App was developed in the development branch, (2) the code was then pushed from development branch to testing environment. (3) The code was tested in the User Acceptance Testing (UAT) Daily environment, and (4) once approved, code was pushed out to production environments (FDA-SCI-HIVE, FDA-HIVE and Public-HIVE which are different HIVE installations at FDA and GW). (5) New code was tested and verified on each environment, with the tester submitting a report to the developer, who (6) addresses bugs in the development branch.

### CGC BCO App

#### Platform

The CGC (www.cancergenomicscloud.org) is a collaborative computational platform that enables researchers to conduct analyses with over 3 PB of data generated through government funding. See Supplementary Materials Section I for more information on the CGC.

#### Tool

Seven Bridges developed a suite of open-source software for BCO generation within their cloud-based platforms to facilitate the communication of complex bioinformatics workflows for regulatory review. End users of the software can access an interactive web application named the CGC BCO App. The BCO App has been deployed on the CGC. However, the design also allows platform-independent deploys and serves it as a stand-alone BCO App, as explained in the deployment section below. See Supplementary Materials Section II for further details on the CGC BCO App tool implementation.

### Galaxy

#### Platform

Galaxy (23) is a web-based computational workbench that is used by thousands of scientists across the world to analyze large biomedical datasets such as those in genomics, proteomics and imaging. More than 7300 analysis tools and 100 visualizations have been integrated into Galaxy, and it has been cited more than 8000 times. Using Galaxy’s Web user interface, any scientist, regardless of informatics expertise, can run complex analysis tools or create and execute multitool workflows. Galaxy ensures that all analyses are completely reproducible (24) by recording all analysis details, such as tool versions and parameter settings, and enabling tools/workflows to be rerun as needed.

#### Tool

BCOs build on existing open standards to maximize utility and extensibility. By creating BCO capabilities in a popular and easy to use bioinformatics platform such as Galaxy (https://galaxyproject.org), researchers will be empowered to automatically create, store, search and run BCOs for data-intensive analyses allowing re-analysis and dissemination of data and knowledge. We extended the Galaxy API (25) to allow the export of Galaxy ‘workflow invocations’ (i.e. realizations of a computational pipeline) in BCO format.

### BCO Portal

#### Tool

Complementary to the previously described platform-dependent BCO editing tools, the BioCompute Portal (previously BCO Editor) is an open-source framework designed to facilitate the creation and documentation of NGS analytical workflows in the form of BCOs. Beyond local storage, BCOs are generally stored on the platform on which the workflow was executed (i.e. CGC, HIVE, etc.) and/or GitHub. In a web application environment, the BioCompute Portal creates BCOs based on the BioCompute schema outlined in the BioCompute Specification Document. The BioCompute Portal Web application (BCO Portal; https://portal.aws.biochemistry.gwu.edu/) allows non-computational biologists to create, store and share their BCOs in a secure, user friendly manner. This is especially useful for users creating knowledge bases where there are several manual review steps. Users register for an account at the website; once approved, users can create and store BCOs and browse and download all open-access BCOs. Researchers can search for existing workflows, upload, create, modify, store and share novel BCOs. The BCO Portal User tutorial is available at: https://github.com/biocompute-objects/bco_editor/blob/main/docs/user_tutorial.md.

**Figure 2.**
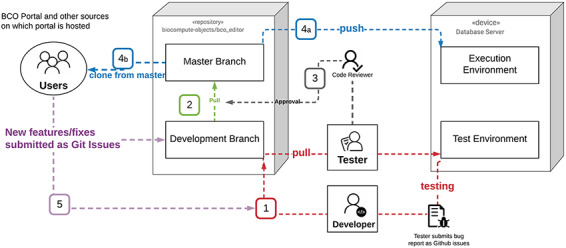
BCO portal deployment diagram.

The BCO Portal is a framework for allowing users to document and store BCOs generated by any tool compliant with the BCO standard. The BCO Portal is platform agnostic, allowing for BCOs generated in platform-specific environments to be collected within a common BCO database. The goal of a common database is to facilitate a wide range of researchers across government, academia and industry to share validated workflows. Thus, the BCO Portal is a unique resource for FAIR compliant workflow exchange. See Supplementary Materials Section II for further details on BCO Portal implementation. The BCO Portal deployment diagram can be found in Figure 2.

(1) Testing: the developer worked in the Development environment, and once a new version was ready for testing it was pushed from the development branch in GitHub to the Test Environment. In the event testing of the new code generated a bug report, a GitHub issue was created for each bug. The developer then addressed the GitHub issues in the development environment and closed issues with commits to the development branch, which was then tested again. This cycle repeated until no issues remained. (2) Pull Request: once the test code was free of bugs the developer assigned a version, rebased and made a pull request from the development branch to the master branch (the master was set as a protected branch). (3) Approval: all pull requests required the review and approval of two administrators, who checked the commit history, bug fixes, etc. Once a pull request was approved, the new version was assigned the appropriate number and listed as a new release. (4) a. The new stable release was pushed to the Execution Environment (biocomputeobject.org) and b. became available for others to clone into their own environments. (5) New features and potential fixes were submitted to GitHub as issues. These issues were triaged and if warranted, incorporated into the next round of development.

### BioCompute tool evaluation

#### Establishing a system for evaluation of BCO tools

**Figure 3.**
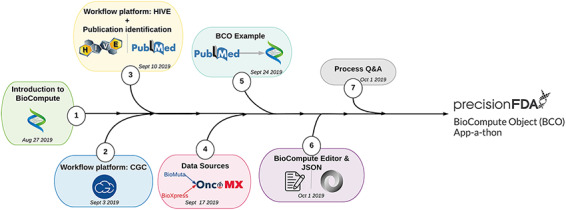
User BCO training process.

In an effort to both broaden exposure to BioCompute and to bolster user understanding of communicating analysis pipelines, BioCompute was integrated into bioinformatics course curricula. We established a new unit as part of the Introduction to Bioinformatics course at the George Washington University. The training module introduced participants to the concept of BioCompute, offered hands on exercises with two genome analysis platforms (HIVE and CGC), introduced the concept of data sources (through the OncoMX, BioMuta and BioXpress knowledge bases (26) (https://www.oncomx.org/)), described the process of finding a suitable publication with an associated open source pipeline and introduced the JSON language. Figure 3 provides an overview of the user BCO training process. Each of the tools developed for creating BCOs were accompanied by documentation materials and authored training materials. These training materials were initially presented as lecture materials. Participant evaluations allow us to assess participant mastery of the BCO material and BCO tool experiences. See Supplementary Materials Section III for thorough protocol on unit design, user documentation and instructions that can be used to replicate the tools training.

The BioCompute training unit (1) introduced participants to the concept of BioCompute, (2) offered hands-on exercises with two genome analysis platforms (HIVE and CGC), (3) familiarized participants with a prominent literature resource (PubMed), (4) introduced the concept of data sources [through the OncoMX knowledgebase (https://www.oncomx.org/)], (5) described the process of finding a suitable publication with an associated open source pipeline, (6) introduced the JSON language as a suitable segue into training users on the BCO Portal (previously BCO Editor) and (7) provided users with a Q&A opportunity prior to final submission of BCOs to the precisionFDA BCO App-a-thon.

#### PrecisionFDA BCO App-a-thon

Participants submitted their generated BCOs to the beginner track of the precisionFDA BCO App-a-thon. PrecisionFDA is a high-performance computing platform where large datasets can be hosted, managed and analyzed in a secure environment. PrecisionFDA connects experts, citizen scientists and scholars from around the world and provides them with a library of tools and reference data. The platform uses a crowdsourcing model and challenges users to develop innovations that inform the science of precision medicine and the development of regulatory science. Those who have registered to participate in this pFDA challenge come from many diverse backgrounds and perspectives, including NGS-based test and software providers, standards-making bodies, pharmaceutical & biotechnology companies, sequencing instrument manufacturers, healthcare providers, academic medical centers, consumers or patients, research consortia and government agencies.

One of the most popular outward facing features of pFDA is the challenge framework. This allows -omics challenges to be conducted in a public facing high-performance computing environment. These challenges focus experts around the world to consider common problems in evolving areas of -omics science such as genomics and proteomics. Challenges are published with instructions, inputs and expectations and typically run for a few months, allowing industry and academic participants to submit results, after which the submissions are evaluated and top performers are identified.

The pFDA team, the Center for Biologics Evaluation and Research (CBER) FDA and George Washington University collaborated to provide a new challenge to highlight BCOs, an evolving standard that provides identification and operational information about bioinformatics pipelines and how they are used. The BioCompute Challenge on pFDA focused on creating software to facilitate the creation and certification of BCOs. More information on the pFDA challenge can be found at https://precision.fda.gov/challenges/7.

## Results

After the development of tools for the creation of BCOs, a BCO Tool Evaluation unit was designed to evaluate the tools themselves and the sufficiency of existing documentation; the result of these efforts are the BCOs submitted to the pFDA challenge and feedback obtained on the BioCompute Specification, platform training materials and app manuals. Each of the developed tools for creating BCOs were accompanied by documentation materials and authored training materials.

### HIVE

#### HIVE BCO App features

The HIVE built-in tool to create BCOs automatically gathers computational information and builds the framework of a BCO based on any computations a user chooses. HIVE populates the appropriate information into the BCO by pulling computational data from the server (computation type, tool type, parameters, input objects and the corresponding files generated as outputs) and displays the information in multiple tables. Once displayed, users are unable to modify the Pipeline Computations section for preservation. Pipeline steps are sorted according to the sequence in which the computations were run. Once all fields are complete, users are able to create the BCO and download a valid JSON. Extra information captures any information not contained within the first two categories and includes the Extension Domain or connecting to external resources not defined in the BioCompute specification. Though the BCO type archived in the system contains specific information, not necessarily the entire BCO, HIVE is able to generate a complete BCO based on this information.

The HIVE platform was introduced to the participants through a presentation that briefly discussed HIVE’s interface, algorithms, visualizations, performance and different genome sequencing technologies, specifically RNA-seq software and pipelines. To familiarize them with RNA-seq workflows, the lecture included a live demo of a differential expression analysis in HIVE with publicly available data. Following the lecture, 23 participants performed variant calling analysis in HIVE as a follow-up exercise.

### Cancer genomics cloud

#### CGC BCO app features

The features in the CGC BCO App span anticipated use cases for generating BCOs in academic, industry and government settings. The app includes an (optional) login module, which allows the application administrators to configure user access control and permission management. After signing into the app, the user can browse and search definitions of specific domains or fields from an interactive table version of the BCOs specification under the ‘Help’ tab. The main features of the CGC BCO include the BCO Creator, BCO Composer, BCO Validator and the BCO Browser, with each feature implemented as a separate tab in the graphical user interface.

The content of the CGC training module covered the use of BCOs in bioinformatics and using the CGC to develop and document workflows. The Seven Bridge team presented a complementary lecture, titled ‘BioCompute Objects as a Framework for Uniting Advances in Workflow Portability and Reproducibility’ that covered an introduction to the CGC and an introduction to generating BCOs on the CGC. This was followed by a hands-on workshop on how to run an RNA-seq workflow on the CGC. The participants received material to prepare them in getting started on the CGC and completed a manual on setting up the RNA-seq workflow as an independent analysis exercise. As a follow-up assessment, the participants were also instructed on how to generate BCOs from an existing workflow. Ten participants completed the assessment, successfully generated a BCO and provided feedback on the app and user flow. The CGC team communicated with the GW (George Washington University) participants through email and video chat to resolve issues. Nearly all participants were able to troubleshoot issues through electronic communication resulting in successful BCO creation.

New CGC training materials were developed specifically for novice tool users that focus on the basics of the CGC, the use of BCOs and development of an RNA-seq workflow for the training module at GW. Also assembled was a new quickstart guide that uses uncontrolled data and a new manual that walks the participants through setting up an RNA-seq workflow. This new material makes the CGC more accessible to new users. Whereas the training CGC training material provides an introduction to CGC concepts, the CGC BCO App User Manual (https://sbg.github.io/bco-app/bco-app-user-manual.pdf) describes practical details on installing, accessing and executing the BCO App. The CGC BCO App User Manual also provides a detailed step-by-step guide for generating BCOs on the CGC.

Users generally reported that the CGC BCO App was easy to use. However, app setup and two critical bugs arising from an older release of Debian/curl on the CGC introduced difficulties for app use. To resolve this, a dedicated server that will host the CGC BCO App is planned and is expected to eliminate start up time. Resolutions that were identified for the two critical bugs will be described in the next version of the CGC BCO App User Manual. A dedicated server hosting the CGC BCO App will also enable app developers to have more control in addressing issues caused by the hosting environment (critical bugs).

### Galaxy

#### BCO galaxy API extension features

The Galaxy BCO API extension takes existing features and aggregates the information needed to populate a BCO. BCO population in Galaxy is 100% automatic, although some fields left are unpopulated as Galaxy is not able to generate them automatically. Instead, the expectation is that the user will complete the BCO via the BCO Portal or other means.

### BCO Portal

#### Tool features

The purpose of the BCO Portal create a BCO even if they have no access to platform-specific tools or knowledge of the standard. It is a form-based system that walks users through the creation of a compliant BCO, indicating required fields. The BCO Portal was designed with five core functions: upload, create, modify, share and store a BCO. To upload an existing BCO, a user will ‘create an object’ and then copy/paste the raw JSON text of an existing BCO. The BCO Portal maps the JSON to the Portal fields, validates the object against the IEEE Schema and creates the BCO to be stored in the BCO Portal database.

BCOs may be submitted via a web-form or as a pre-formatted document in the JSON format. Submitted BCOs are validated for conformance to the BCO JSON Schema and for uniqueness (submission of duplicate BCOs is not allowed). By default, all BCOs are accessible for viewing to all registered users. However, end users often submit BCOs for workflows of a confidential nature, e.g. for a drug submission to a regulatory agency. In such cases, the BCO author has the option of specifying a date range during which the BCO will be hidden from public view. To ensure continuing collaboration, the author can provide read/write or read-only access to other registered users during this embargo period.

Through the storage of BCOs, the BCO Portal is aggregating a database of BCOs that can be utilized as reference for fellow researchers. For example, a student or researcher seeking to know more about an existing pipeline for differential expression analysis using specific RNA-seq tools in a shell environment can search the BioCompute Portal for this and would have access to download the JSON and use it for experimental reference in their own research. A central, platform-independent location to create, edit, share and query novel and existing BCOs is an ideal structure for making publicly available workflow information in the form of BCOs available. The BCO Portal enables users to systematically access and track existing BCO data records that include metadata and QC processes intrinsic to BCO files, thereby promoting computational transparency and appropriate reuse of bioinformatics workflows. Further, an aggregated approach to storing and accessing BCOs make it possible for other platforms to gain access to the repository via API.

### GitHub issues

Over the course of 22 days, 83 GitHub issues (a tracking system for bugs and enhancements) were created in the GW-SMHS-BIOC6223 repository. A significant number of issues (20+ of 83 total issues) were user feedback on the BCO Portal that identified minor and critical bugs in the BCO Portal, ranging from permission levels, checksum error messages and the inability to save objects. Documentation issues were submitted as requests. Additions or modifications to the current BioCompute Specification document were also logged for further discussion. Feedback generated through GitHub will be reviewed and provided to the BioCompute Technical Steering Committee for further review and possible enhancement of the current documentation in the next release of the specification document.

### User experience

Feedback on BCO training practices, resources and BCO creation tools was obtained through three avenues: GitHub issues, extra-credit deliverables and a post-pFDA submission survey. Feedback topics included requests for more tool and platform documentation, bug reporting for CGC, HIVE and BCO Portal and content of Specification v1.3 (particularly related to clarity and breadth of information and to protocol sufficiency).

Throughout the BCO unit, participants were encouraged to leave feedback via GitHub issues. This real-time and actionable feedback system was invaluable as participants were able to provide feedback on a variety of topics/concerns as they were encountering them. For example, a GitHub issue created by a participant who, while creating and modifying her BCO, felt there was a need for more thorough documentation regarding the software prerequisites in the Execution Domain. Her feedback was in response to her group’s discussion regarding inconsistencies found in the review process. Despite adapting the same publication for their BCOs, group members had different software prerequisites recorded and they had been encouraged to discuss the differences and reasons behind them as a group. For clarification, the group consulted the resources available to them, including the BioCompute Specification Document, the BCO Portal tooltips and the Example BCO but were unable to find the information they needed. As a result, the user created a GitHub issue (https://github.com/biocompute-objects/GW-SMHS-BIOC6223/issues/173) with the labels ‘BCO Portal,’ ‘documentation’ and ‘feedback’. The pipeline described in the referenced publication used Trim Galore (http://www.bioinformatics.babraham.ac.uk/projects/trim_galore/) for simultaneous adapter trimming and quality check. One group member had included module details for Cutadapt (27) in pipeline step, as part of the Description domain. In this version, Trim Galore was listed as a software and Cutadapt as the prerequisite. In comparison, another participant referenced Cutadapt in the Execution domain’s Software Prerequisites. The inability to distinguish a particular prerequisite as a pipeline step or platform prerequisite was described in the GitHub issue by the participant as a ‘lack of consistency’ and counterproductive to standardization. Insight from feedback like this is planned to be addressed in a Best Practices document that will offer guidance on practical implementations of BioCompute.

There was also an opportunity for participants to provide feedback and contribute to the software development process. The participants had first demonstrated a basic understanding of workflows, the CGC and BCOs. Participants were asked to translate an existing workflow into a project on the CGC, generate a BCO using the CGC’s BCO App and provide feedback on the process. SB personnel were available to interact with participants throughout the process. Specific instructions (including a list of potential workflows to use on CGC), training documentation and manuals were provided by Dr Dennis Dean (Principal Investigator at Seven Bridges) and Dave Roberson (Seven Bridges Community Engagement Manager) were available as a resource for participants in addition to his development team. The process had three parts: (i) BCO Submission to the pFDA, (ii) BCO Generator App Review and (iii) BCO Standard Recommendation. As the practical application of the BCO standard by inexperienced users had been previously untested, the latter two parts were a call for feedback. Participants submitted a one to two paragraph review of the BCO App deployed on the CGC. The participants submitted an additional one to two paragraph descriptions of changes to the BCO Standard that might make it easier to record and recall workflow information in a structured way.

Additional feedback was collected from the BCO User Experience Survey distributed to the participants post-submission to the pFDA challenge. Table 1 provides a sample of these questions. The survey focused on three core areas: confidence in the training of the BCO Framework, availability of BCO resources/thorough documentation and the platforms/tools used throughout the process: GitHub, HIVE platform, CGC platform and CGC BCO App, BCO Portal (considerations such as ease of use, GUI, etc.). For example, did participants feel comfortable utilizing GitHub as a means of issue resolution in a collaborative environment? How confident are they in their ability to utilize the platforms for workflow and analysis? Training Module survey results can be found in Supplementary Materials Section IV.

**Table 1.** Training module user survey

Question	Answer
How clear were the evaluation unit objectives?	1 (unclear) → 5 (extremely clear)
Did the structure and sequence of the lectures make sense?	1 (not at all) → 5 (very much so)
Did the unit expose you to new knowledge, tools and practices?	Definitely, yes Yes, sort of Not really Definitely not
Of the new knowledge, tools and practices this module taught, how comfortable do you feel using GitHub as a means of code/project management and collaboration?	1 (not at all comfortable) → 5 (extremely comfortable)
Of the new knowledge, tools and practices this module taught, how comfortable do you feel with running computational analysis on HIVE?	1 (not at all comfortable) → 5 (extremely comfortable)
Of the new knowledge, tools and practices this module taught, how comfortable do you feel with running computational analysis on CGC?	1 (not at all comfortable) → 5 (extremely comfortable)
Of the new knowledge, tools and practices this module taught, how comfortable do you feel with generating BioCompute Objects in CGC?	1 (not at all comfortable) → 5 (extremely comfortable)
Of the new knowledge, tools and practices this module taught, how comfortable do you feel with creating BioCompute Objects in the BCO Editor?	1 (not at all comfortable) → 5 (extremely comfortable)
GUI: In your opinion, how user friendly is HIVE’s interface?	1 (not at all) → 5 (extremely)
GUI: In your opinion, how user friendly is CGC’s interface?	1 (not at all) → 5 (extremely)
GUI: In your opinion, how user friendly is BCO Editor’s interface?	1 (not at all) → 5 (extremely)

### User stories

The following user stories describe how integrating BCOs within existing applications are advancing FAIR principals.

#### Knowledge base

GlyGen, an NIH-funded glycoconjugate database leverages the BCO Portal to document data integration pipelines and provide complete transparency and accessibility to its users and collaborators. GlyGen data managers use a separate instance of the BCO Portal to generate a BCO for each integrated dataset in GlyGen. The ease of use of this form-based system allows the data managers to add relevant information into predefined rules and fields efficiently. This information is readily converted into a JSON format which is hosted in the GlyGen database. In addition to the user-friendly interface, the generated GlyGen BCOs can be easily viewed under one domain and searched based on BCO name, contributors or BCO IDs. In addition to maintaining a standardized format, the BCO Portal significantly reduces the GlyGen data managers’ manual effort and time otherwise required to generate a single BCO.

#### Computation

HIVE platform team employs BCOs as communication of internal testing protocols. BCOs have been developed using the HIVE BCO App and the BCO Portal to record routine testing pipelines to evaluate functionality of novel and third party tools within an instance of HIVE hosted on Amazon Web Services (28). Further testing computations and BCO details can be found in Table 2.

**Table 2.** BCOs recording HIVE platform testing protocol

Tool(s)	BCO(s)	Computation	Inputs
HIVE-Hexagon	BioCompute Portal: BCO_026619	HIVE Object ID: 3249	**Read** (paired-end) Name: SRR1004397_1 SRR1004397_2
	GitHub: HIVE-hexagon Test Computation_BCO_026619		HIVE Object ID: 729, 731 **Reference** Name: GrCH38.2 MARCH14–2016
HIVE-Heptagon	BioCompute Portal: BCO_023769	HIVE Object ID: 3260	**Alignment**: SRR1004397_1 against GrCH38.2 MARCH14–2016
	GitHub: HIVE-heptagon_Test_Computation_BCO_023769		HIVE Object ID: 3249
CensuScope	BioCompute Portal: BCO_015623	HIVE Object ID: 3242	**Read**: mgm4461125.3.050.upload.fna 219458
	GitHub: HIVE-CensuScope Test Computation_BCO_015623		HIVE Object ID: 2218 **Reference**: filtered_nt_July_2018 HIVE Object ID: 2242

- Testing Pipeline #1 evaluates Hexagon (29), a sequence alignment tool that allows the user to align reads from a high-throughput experiment to a reference genome. Hexagon was used to align human DNA samples from Whole Exome Sequencing of lung squamous carcinoma (SQCC) patients against human reference genome GRCh38. BCOs were created using the BCO Portal and the HIVE BCO App. These BCOs are freely available in the BCOs GitHub repository (https://github.com/biocompute-objects).
- Testing Pipeline #2 evaluates Heptagon (30), a tool that performs base and SNP-calling for a previously computed alignment and provides quality and noise assessment profiles. Heptagon was used to identify SNPs from the previous Hexagon alignment of Whole Exome Sequencing of lung SQCC patients against human reference genome *GRCh38.* BCOs were created using the BCO Portal and the HIVE BCO App. These BCOs are freely available in the BCOs GitHub repository (https://github.com/biocompute-objects).
- Testing Pipeline #3 evaluates CensuScope (31), a tool designed and optimized for the quick detection of the components of a given NGS metagenomic dataset, providing users with a species-level composition of a given sample. CensuScope was used to map a human gut microbiome sample (sourced from MG-RAST) against FilteredNT to view the sample’s taxonomic composition. BCOs were created using the BCO Portal and HIVE BCO App. These BCOs are freely available in the BCOs GitHub repository (https://github.com/biocompute-objects).

#### Regulatory submission

To investigate how a BCO would supplement the submission of a Phase II, randomized, open-label clinical trial that evaluated the efficacy and safety of a combination of HCV1a drugs, the 2019 BCO Proof of Concept project (32) started as a collaboration between GW, FDA and DDL. The project objective was the replication of a clinical trial submission with mock clinical data from the FDA to confirm if BCO facilitates the regulatory agency submission process by investigating potential discrepancies found between data analysis pipelines. The DDL Athena NGS pipeline has been used to test more than 20 000 samples from clinical trials involving hepatitis C virus (HCV), hepatitis B virus (HBV), cytomegalovirus (CMV), respiratory syncytial virus (RSV) and SARS-CoV-2 (33). While there are clear guidelines on how to report NGS data to FDA, there is no standardization on how to describe the computational workflow used during the data analysis. A BCO would not only help to clearly communicate with the regulatory agencies but would also be an aid to show the high-quality sequencing results appropriately. Additionally, with the BCO, sponsors such as DDL can generate the necessary submission documentation faster and therefore reduce internal costs. Two separate analyses were executed: one to simulate a pharmaceutical submission to the FDA and another to simulate the FDA review. BCOs from the process were generated for communication of process and comparison of result.

## Discussion

This paper introduces four novel tools for generating BCOs: BCO Portal, HIVE BCO App, CGC BCO App and Galaxy BCO API Extension. The stand-alone BCO Portal supports multi-platform workflows and provides a universal method to BCO creation and storage. The tools used in the context of a platform—CGC, HIVE and Galaxy—are designed to semi-automate the process of generating a BCO by extracting pertinent information from workflows native to the specific platform. These platform-specific tools generate a formatted BCO JSON object by extracting pipeline steps, platform information, data locations and parameters, while allowing a user to manually enter provenance and metadata information if not already extracted. As a key feature of BioCompute is interoperability (34), these four BioCompute tools were developed with the capability to ingest and store the same BCO. It is envisioned that other platforms may also integrate support for the standard, enabling researchers to more easily collaborate across environments, or to communicate workflows to a central authority like the FDA or to a publisher.

To evaluate the BCO Portal and CGC BCO App, bioinformaticians at the George Washington University built and curated BCOs from published workflows using the BCO creation tools introduced in this paper, and the completed BCOs were subsequently submitted to the precisionFDA BCO Challenge (21). Prior to pFDA challenge submission, each BCO was submitted for review to a BioCompute technical assistant in the GitHub repository, allowing rapid feedback from the BCO reviewer through the built-in issue-tracking system, and leveraging reviewer metadata intrinsic to the standard. This review process simulated a real quality and integrity review and established an official reviewer that was included in the BCO provenance domain. This user evaluation of the BioCompute tools via training module served two purposes: indicating usability of tools and furthering adoption initiatives.

The primary purpose of user evaluation was to indicate the usability of the tools and intelligibility of the BioCompute standard itself; this evaluation highlighted potential challenges within the BCO creation process. These challenges led to further development in the form of tool bug fixes and the introduction of new features. Assessing these tools in this manner engaged users and benefitted tool developers by providing specific areas their tools can be improved.

User evaluation also resulted in an introduction to the process of testing recently developed tools and further developing an emerging standard for novice bioinformaticians. Building BCOs from published work provided users: (i) exposure to collaborative workflows and the process of building bioinformatics pipelines, (ii) hands-on experience participating in a review process of a public repository, (iii) exposure to tool development as testers and (iv) a portfolio item in the form of a pFDA challenge submission. Users had the opportunity to work with biotechnology professionals active in the development of BioCompute, imparting upon them a greater understanding of the interaction between academic, industry and government institutions. Learning to navigate new code bases (bugs) by engaging directly with developers is a learning experience most novice bioinformaticians are not exposed to. Users who reported and discussed challenges in generating BCOs developed a strong understanding of both the tools and the standard. These novice non-informatician biologists ultimately produced actionable feedback as participants in the testing and development of the tools and training materials that will further enhance the BCO Specification and likely accelerate the acceptance of the BCO standards.

### FAIR compliance

As BCOs are compliant with FAIR principles, the specification and schema contain features designed to make the encapsulated workflows and datasets findable, accessible, interoperable and reusable software, datasets and workflows. Each BCO provides execution data with corresponding scripts and script drivers necessary for workflow reproducibility, and data location accessibility requirements are transparent.

In addition to the adherence to FAIR data standards, the BioCompute Framework aligns with USFDA guidelines for Database Procedures and Operations (35); it enables transparency and public accessibility of data sources and standard operating procedures, in addition to ensuring secure version control.

### Database applications

Beyond bioinformatics analysis, the BioCompute framework has successfully been applied to knowledge base data integration. Over the last two years, GlyGen (36), an NIH-funded glycoinformatics project, has generated BCOs for over 200 individual datasets (https://data.glygen.org/). Each individual BCO not only provides complete transparency of its data integration process to its authors, contributors and users but also includes detailed information of its data usability, data modification, versioning, keywords and quality control pipelines. Using the I/O (Input/Output) and execution domains, GlyGen provides the input, output (validated and failed) and script files to allow easy reproducibility and replicability by its users. Through BCO’s predefined fields and rules, GlyGen is able to document different data-specific workflows in a standardized format effectively. The generated BCOs are freely accessible for browsing and downloading through the GlyGen data portal (https://data.glygen.org/) under license CC BY 4.0. Similar BCOs also are available for the OncoMX knowledge base (https://data.oncomx.org/) (37).

In summary, in addition to the evaluation of the BCO framework, the process has been an effective method for evaluating BCO creation tools (CGC BCO App and BCO Portal) and training users to be resourceful in tool development. The BioCompute tools this paper presents make it easier to create tools for an emerging standard and are available prior to release. A preliminary review of the feedback provided identified potential changes to the BioCompute Specification Document, additions to the CGC BCO App training materials and BCO Portal modifications. Future tool releases will have increased usability due to tool enhancements and documentation revisions recommended by users.

### Future applications

Future work will build on these tools, such as by building databases and repositories of validated BCOs, integrating them into relevant government and academic systems and working with private sector participants to help integrate the standard into their existing platforms, based on the work presented here, to expedite communication. The BCO-based system could evolve to become a formalized mechanism of communication, such as by the Drug Master File or as part of its own section in an application, to government agencies like the FDA, USPTO (United States Patent and Trademark Office), CMS (Center for Medicare and Medicaid Services), CDC (Center for Disease Control and Prevention), EPA (Environmental Protection Agency) and others.

## Conclusion

Emerging data analysis challenges include increasing dataset size and complexity that cannot be practically copied for analysis due to slow transfer rate, archival maintenance, privacy concerns and data ownership restrictions. As datasets grow very large, there is a growing interest in bringing computations to data rather than the other way around, such as through cloud service providers partnering with the STRIDES (Science and Technology Research Infrastructure for Discovery, Experimentation, and Sustainability) program. Consequentially, there is a need to document NGS workflow analyses, including NGS data provenance across a range of computational environments including computational platforms that support genomic analysis, with the goal of ensuring that these analyses can be replicated in a variety of environments. We further posit that detailed NGS documentation requires a clear communication of NGS analysis (workflows and data usage) that is both human and machine readable. The BioCompute standard, IEEE 2791-2020, aims to fill this need to clearly communicate NGS analysis workflows and has the potential for accelerating research (34). BCO tools allow researchers to create BCOs that adhere to the community-developed BioCompute Specification to encode pertinent information to record data provenance, facilitate regulatory review and improve reproducibility of results, and they allow them to do so quickly and easily, without needing to learn the standard.

In summary, in addition to the evaluation of the BCO framework, the process has been an effective method for evaluating BCO creation tools (CGC BCO App and BCO Portal) and training users to be resourceful in tool development. The BioCompute tools this paper presents make it easier to create tools for an emerging standard and are available prior to release. A preliminary review of the feedback provided identified potential changes to the BioCompute Specification Document, additions to the CGC BCO App training materials and BCO Portal modifications. Future tool releases will have increased usability due to tool enhancements and documentation revisions recommended by users.

## Supplementary Material

baab008_Supp

## References

[1] Simonyan,V., Chumakov,K., Dingerdissen,H. et al. (2016) High-performance integrated virtual environment (HIVE): a robust infrastructure for next-generation sequence data analysis. *Database (Oxford)*, 2016, 1–16. 10.1093/database/baw022 PMC4795927 26989153

[2] Simonyan,V., Mazumder R. (2014) High-Performance Integrated Virtual Environment (HIVE) Tools and Applications for Big Data Analysis. *Genes (Basel)*, 5, 957–981. 25271953 10.3390/genes5040957 PMC4276921

[3] Lau,J.W., Lehnert,E., Sethi,A. et al. (2017) The cancer genomics cloud: collaborative, reproducible, and democratized – a new paradigm in large-scale computational research. *Cancer Res.*, 77, e3–e6. 29092927 10.1158/0008-5472.CAN-17-0387 PMC5832960

[4] Jalili,V., Afgan,E., Gu,Q. et al. (2020) The Galaxy platform for accessible, reproducible and collaborative biomedical analyses: 2020 update. *Nucleic Acids Res.*, 48, W395–W402. 32479607 10.1093/nar/gkaa434 PMC7319590

[5] *Genomic Knowledge Standards* . (2017) https://www.ga4gh.org/work_stream/genomic-knowledge-standards/ (31 December 2020, date last accessed).

[6] Watkins,M., Rynearson,S., Henrie,A. et al. (2019) Implementing the VMC specification to reduce ambiguity in genomic variant representation. *AMIA Annu. Symp. Proc.*, 2019, 1226–1235. 32308920 PMC7153148

[7] Hl7. (2014) *FHIR Specification FHIR v0.0.82*. http://hl7.org/fhir/ (31 December 2020, date last accessed).

[8] Amstutz,P., Chapman,B., Chilton,J. et al. (2016) Common Workflow Language, v1.0 Common Workflow Language (CWL) Command Line Tool Description, v1.0.

[9] OpenWDL. (2018) Workflow Description Language. https://openwdl.org/#three (31 December 2020, date last accessed).

[10] Koster,J. and Rahmann,S. (2012) Snakemake—a scalable bioinformatics workflow engine. *Bioinformatics*, 28, 2520–2522. 22908215 10.1093/bioinformatics/bts480

[11] Seqera Labs. (2020) *Nextflow - A DSL for Parallel and Scalable Computational Pipelines*. https://www.nextflow.io/ (31 December 2020, date last accessed).

[12] Carragáin,E.Ó., Goble,C., Sefton,P. et al. (2019) A lightweight approach to research object data packaging.

[13] Kanwal,S., Khan,F.Z., Lonie,A. et al. (2017) Investigating reproducibility and tracking provenance – a genomic workflow case study. *BMC Bioinform.*, 18, 337. 10.1186/s12859-017-1747-0 PMC5508699 28701218

[14] *IEEE 2791–2020 - IEEE Standard for Bioinformatics Analyses Generated by High-Throughput Sequencing (HTS) to Facilitate Communication* . (2020) https://standards.ieee.org/standard/2791-2020.html (31 December 2020, date last accessed).

[15] Simonyan,V., Goecks,J. and Mazumder,R. (2017) Biocompute Objects-A Step towards Evaluation and Validation of Biomedical Scientific Computations. *PDA J. Pharm. Sci. Technol*., 71, 136–146. 27974626 10.5731/pdajpst.2016.006734 PMC5510742

[16] BCO_Specification . (2018) *Repository for Support of the IEEE 2791–2020 Standard*. https://github.com/biocompute-objects/BCO_Specification (6 May 2020, date last accessed).

[17] Pezoa,F., Reutter,J.L., Suarez,F. et al. (2016) Foundations of JSON schema. In: *25th International World Wide Web Conference, WWW 2016*, International World Wide Web Conferences Steering Committee, Montréal Québec, Canada, pp. 263–273.

[18] Federal Register . (2020) *Electronic Submissions; Data Standards; Support for the International Institute of Electrical and Electronics Engineers Bioinformatics Computations and Analyses Standard for Bioinformatic Workflows* https://www.federalregister.gov/documents/2020/07/22/2020-15771/electronic-submissions-data-standards-support-for-the-international-institute-of-electrical-and (8 January 2021, date last accessed).

[19] Xiao,N., Koc,S., Roberson,D. et al. (2020) BCO app: tools for generating BioCompute Objects from next-generation sequencing workflows and computations. *F1000Research*, 9, 1144. 10.12688/f1000research.25902.1 PMC7702177 33299553

[20] Hornik,K. (2012) The comprehensive R archive network. *Wiley Interdiscip. Rev. Comput. Stat.*, 4, 394–398.

[21] Stephens,S.H., King,C.H., Watford,S. et al. (2020) Strengthening the BioCompute standard by crowdsourcing on PrecisionFDA. *bioRxiv*, 2020.11.02.365528.

[22] Wilkinson,M.D., Dumontier,M., Aalbersberg,I.J. et al. (2016) Comment: the FAIR guiding principles for scientific data management and stewardship. *Sci. Data*, 3, 160018. 10.1038/sdata.2016.18 PMC4792175 26978244

[23] Afgan,E., Baker,D., Batut,B. et al. (2018) The Galaxy platform for accessible, reproducible and collaborative biomedical analyses: 2018 update. *Nucleic Acids Res.*, 46, W537–W544 29790989 10.1093/nar/gky379 PMC6030816

[24] Grüning,B., Chilton,J., Köster,J. et al. (2018) Practical computational reproducibility in the life sciences. *Cell Syst.*, 6, 631–635. 29953862 10.1016/j.cels.2018.03.014 PMC6263957

[25] Sloggett,C., Goonasekera,N. and Afgan,E. (2013) BioBlend: automating pipeline analyses within Galaxy and CloudMan. *Bioinformatics*, 29, 1685–1686. 23630176 10.1093/bioinformatics/btt199 PMC4288140

[26] Dingerdissen,H.M., Torcivia-Rodriguez,J., Hu,Y. et al. (2018) BioMuta and BioXpress: mutation and expression knowledgebases for cancer biomarker discovery. *Nucleic Acids Res*, 46, D1128–D1136. 30053270 10.1093/nar/gkx907 PMC5753215

[27] Martin,M. (2011) Cutadapt removes adapter sequences from high-throughput sequencing reads. *EMBnet.J.*, 17, 10.

[28] Amazon . (2015) About AWS. https://aws.amazon.com/about-aws/ (15 October 2019, date last accessed).

[29] Santana-Quintero,L., Dingerdissen,H., Thierry-Mieg,J. et al. (2014) HIVE-hexagon: high-performance, parallelized sequence alignment for next-generation sequencing data analysis. *PLoS One*, 9, e99033. doi: 10.1371/journal.pone.0099033 PMC4053384 24918764

[30] Simonyan V., Chumakov,K., Donaldson,E. et al. (2017) HIVE-heptagon: a sensible variant-calling algorithm with post-alignment quality controls. *Genomics*, 109, 131–140. 28188908 10.1016/j.ygeno.2017.01.002

[31] Shamsaddini,A., Pan,Y., Johnson,W.E. et al. (2014) Census-based rapid and accurate metagenome taxonomic profiling. *BMC Genomics*, 15, 918. doi: 10.1186/1471-2164-15-918 PMC4218995 25336203

[32] Hadley,C., King,S., Keeney,J. et al. (2020) Communicating regulatory high throughput sequencing data using BioCompute Objects disclaimer. *bioRxiv*, 2020.12.07.415059. 10.1016/j.drudis.2022.01.007 35077912

[33] Diagnostic Laboratory (DDL). (2016) *Bioinformatics - DDL Diagnostic Laboratory*. https://www.ddl.nl/bio-informatics/#athena-virology-pipeline (8 January 2021, date last accessed).

[34] Alterovitz,G., Dean,D., Goble,C. et al. (2018) Enabling precision medicine via standard communication of HTS provenance, analysis, and results. *PLoS Biol.*, 16, e3000099. 10.1371/journal.pbio.3000099 PMC6338479 30596645

[35] FDA . Use of public human genetic variant databases to support clinical validity for genetic and genomic-based in vitro diagnostics. https://www.fda.gov/regulatory-information/search-fda-guidance-documents/use-public-human-genetic-variant-databases-support-clinical-validity-genetic-and-genomic-based-vitro (15 October 2019, date last accessed).

[36] York,W.S., Mazumder,R., Ranzinger,R. et al. (2019) GlyGen: computational and informatics resources for glycoscience. *Glycobiology*, 30, 72–73. 10.1093/glycob/cwz080 PMC7335483 31616925

[37] Dingerdissen,H.M., Bastian,F., Vijay-Shanker,K. et al. (2020) OncoMX: a knowledgebase for exploring cancer biomarkers in the context of related cancer and healthy data. *JCO Clin. Cancer Inform.*, 4, 210–220. 32142370 10.1200/CCI.19.00117 PMC7101249

