# Supplementary material for: Bioinformatics tools developed to support BioCompute Objects
Source: Database (Oxford). 2021 Mar 30;2021:baab008. doi: 10.1093/database/baab008 (PMC8009203; doi:10.1093/database/baab008)
Supplement: baab008_Supp [file baab008_supp.zip › Supplemental Materials.docx]

# Supplemental Materials

1. Platform Details

HIVE Platform

Devised specifically for the storage and analysis of a myriad of extra-large biomedical data, HIVE has been used to accommodate mass spectrometry files, confocal microscopy images, post-market surveillance data, medical recall data, and many others. There is a common web portal and an API-driven website which allows users to fetch and load data whether directly from the user machine or from external data sources (public sources or sequencing centers to which samples were sent). Large data from diverse sources such as the Sequence Read Archive (SRA)(38) and GenBank(39) can be fetched using the SRA accession numbers and GenBank identifiers; HIVE preserves the datasets in a distributed storage library, seamlessly linking to computational analysis with complete traceability. To address the growing number of data types, HIVE houses a honeycomb database map which allows researchers to design the data types and populate them themselves. Today there are multiple deployments of HIVE: FDA-HIVE (a regulatory analytics platform at the FDA), FDA-SCI-HIVE (a scientific research production platform at FDA), and Public-HIVE (a public instance allocated for public consumption at GW for researchers who want to collaborate, develop new algorithms, or solely use for their own purposes).

CGC Platform

The CGC is a cloud-based microservice architecture system that includes services that support computations, visualizations, data, metadata, SDK, API, and administration. Core to the CGC is the execution of a computational workflow written as a Common Workflow Language Workflow(8). The CGC public gallery contains 400 workflows and apps across many NGS subdomains. Apps allow users to quickly port command-line tools to the CGC. The CGC is able to schedule CWL workflows across multiple cloud instances as specified within the CWL, allowing for an efficient execution on the cloud without the need for user intervention. The recent addition of memoization allows for submitted tasks to recover from periodic, but expected, communication failures between computational nodes. The CGC infrastructure supports scalable computations, reproducible computation, and analysis portability by design with the goal of supporting FAIR principles(22). The CGC infrastructure is an extendable computational platform with notable extensions including support for Jupyter Notebook (<https://jupyter.org/)> and RStudio Server; facilitating interactive analysis of large-scale data analysis hosted on the cloud. The CGC provides an API interface with Python, Java, and R bindings that supports the same features provided by the graphical user interface. CGC users report that their research benefits by the availability of large genomics datasets generated with the support of the National Cancer Institute. The Cancer Genomic Cloud hosts the Human Cell Atlas (HCA)(40), Personal Genome Project UK (PGP-UK)(41), The Cancer Imaging Archive (TCIA)(42), the Clinical Proteomic Tumor Analysis Consortium (CPTAC)(43), the Simons Genome Diversity Project (SGDP)(44), Cancer Cell Line Encyclopedia (CCLE)(45), The Cancer Genome Atlas (TCGA)(46), and the Therapeutically Applicable Research to Generate Effective Treatments (TARGET) dataset(47).

Galaxy Platform

Galaxy provides an advanced Graphical User Interface (GUI) both for composing and running genomic data analysis pipelines, in addition to the Toolshed(48) for downloading and installing a range of commonly used bioinformatics software. Additional features include easy deployment of command-line bioinformatics tools through the GUI, and having a pipeline execution engine(49) in the backend, providing resilience, high-performance and scalability. Copying data analysis pipelines across Galaxy installations at different laboratories is easy through XML descriptor files (http://www.w3.org/xml), but the bioinformatics tools that make up each pipeline need to be re-installed at each location.

1. Tool Implementation

### HIVE BCO App

Source

The HIVE BCO app was developed using the React framework (https://reactjs.org/) and Ant Design library (<https://ant.design/docs/react/introduce>). The React.js front end JavaScript framework was used to build the user interface (UI) because of this schema’s ease of use, reusable components, virtual DOM, and substantial user community. Ant Design (Antd), a components library for ReactJS, was the main third-party library used to build forms, buttons, tabs, etc. Antd is an ideal library because of its seamless integration into React, many components that work well with each other, and responsive development team. The project was built using the git versioning tool to track all the changes to the code. Because the BCO tool was developed inside FDA, the source code will not be available to the public.

#### Deploy

Once the HIVE BCO app was developed, the code was pushed to a testing environment and tested for functionality issues, bugs, and design flaws. Once all concerns were addressed, the app was rolled out into production, with the ability to roll back in the event of any major issues. Currently, the HIVE BCO app is included in HIVE Version 2.3. This version has undergone 2 releases. In early 2019, release 1 was developed, pushed to SCI-HIVE (research use) and regulatory HIVE as well as pushed to GW HIVE for testing by the GW HIVE team. Issues found were reported and addressed in preparation for a live demo during the 2019 BioCompute Workshop hosted at the FDA. Release 2 is currently in development; once complete, it will follow the same deployment plan (Figure 2 HIVE BCO App Deployment Diagram). Development and testing were each carried out by separate individuals.

### CGC App

#### Source

The development team implemented the CGC BCO app with the R web framework Shiny (<https://shiny.rstudio.com/).> The CGC BCO app (<https://github.com/sbg/bco-app>) integrates the R packages *biocompute* and *tidycwl,* which serve as the app’s primary functional components. The *biocompute* package is the Seven Bridges implementation of the BioCompute Specification Document. The package offers the capabilities to compose, validate, and convert BioCompute Objects with R. The *tidycwl* package can read, parse, and render Common Workflow Language tools and workflows in R. The tidyverse design principles (<https://principles.tidyverse.org/>) motivated the design of the two packages with the goal of facilitating package reuse. The source code repositories for the web application and the R packages described above are available from the Comprehensive R Archive Network (CRAN)(20) and the Seven Bridges GitHub organization page (<https://github.com/sbg/>), licensed under AGPLv3.

#### Deploy

The CGC BCO app design enables multiple deployment options that will support multiple testing and production use cases. Currently, the CGC BCO app supports self-managed local deployment, self-managed cloud deployment, and fully-managed cloud deployment. The CGC BCO app was deployed on the Cancer Genomics Cloud with the self-managed cloud deployment option, functionally serving as a platform-dependent app for the work presented in this paper.

### Galaxy

#### Source

The Galaxy project source code is available via GitHub at <https://github.com/galaxyproject/galaxy/>. Contributions to the code base from the general public are welcomed. The BCO additions to the API were originally deployed at <https://galaxy.aws.biochemistry.gwu.edu/>, but have since been incorporated into the main Galaxy codebase.

#### Deploy

The deployment of the BCO Galaxy instance is served using an AWS EC2 instance, but the source code can be deployed via GitHub on any server. Detailed instructions for deploying Galaxy are available at https://galaxyproject.org/admin/get-galaxy/.

### BCO Portal

#### Source

The BCO Portal source code encapsulates two distinct pieces: a frontend UI (user interface) and a backend application programming interface (API). The frontend is built in React, allowing for consistent and modular functionality. The backend is built using Django (https://www.djangoproject.com/), the well-known web server library for Python. Django is connected to an instance of MongoDB (Mongo; [https://api.mongodb.com](https://api.mongodb.com/)). To separate the API from the web server, allowing for tighter security, nginx (<https://nginx.org/>) and gunicorn (<https://gunicorn.org/>) are used in a reverse-proxy. Source code for the BCO Portal is available at <https://github.com/biocompute-objects>.

#### Deploy

The main deployment of the BCO Portal is served using an AWS EC2 instance, but the source code can be deployed on any standard Linux distribution. The portal was first implemented and tested in a test environment; tested code was ported to the final production environment via the BioCompute GitHub repository (Figure 3 BCO Portal Deployment Diagram). The Linux flavor employed for portal.aws.biochemistry.gwu.edu is CentOS. Instructions for installing the BCO Portal on a similar virtual machine are available at https://github.com/biocompute-objects/bco_editor/blob/main/docs/centos.md. Future development plans include further refinement of the frontend UI and API code bases, standardizing documentation, a user interface upgrade, token/session/key-based securitization, BCO regex search capabilities, and dockerizing the API so that users who create BCOs on platforms other than the Portal are able to take advantage of its functionality.

1. Unit design: Establishing a system for evaluation of BCO tools

In an effort to both broaden exposure to BioCompute and to bolster participant understanding of communicating analysis pipelines, BioCompute was integrated into bioinformatics course curricula at The George Washington University. In addition to introducing participants to the concept of BioCompute and the practical application of cloud computing, users learned the concept of organizing data in meaningful ways by building JSON objects out of artificial data, and then exploring the ways in which their JSON object might be parsed for downstream use. This exercise introduced best practices, standardization, the concept of a schema, and was a natural segue for teaching BioCompute.

The final stage of the "Introduction to Bioinformatics” unit dedicated substantial time to leading participants through the process of documenting an entire pipeline. Through this process, participants became familiar with the concept of pipelines generally, including recapitulation of a high-level explanation of the pipeline, a granular, stepwise description of the pipeline, and an acknowledgement of all necessary prerequisites and input files.

The final step of the process was formal submission to the FDA via the Beginner Track of the BCO Challenge (<https://precision.fda.gov/challenges/7>) on the pFDA platform. Because the BCO Challenge is meant to attract both novice and advanced programmers, and to provide exposure to those unfamiliar with BioCompute, it is an ideal mechanism for user participation. Submission to the pFDA platform supported participant education by 1. Encouraging direct engagement with the FDA, 2. Building a BCO from an open sourced, published pipeline that can be showcased as a portfolio item, and 3. Adding greater visibility to the participants’ work.

In addition to broadening exposure to – and feedback for – the BioCompute specification, this unit was designed to give participants a powerful, hands-on introduction to the general pipeline concepts, and to some of the most common tools used in genome analysis. Contributors were required to pick a cancer-related publication that used an RNA-seq pipeline, and to capture the details of the pipeline in a way that made it understandable to a potential reviewer. Along the way, they got hands-on training with two genome analysis platforms with direct engagement with platform developers and administrators, developed an intuitive sense for the meaning and purpose of various aspects in a pipeline, and received exposure to working collectively on an online repository. See supplemental materials for user documentation and instructions that can be used to replicate the training.

### Project management and collaboration through GitHub

#### Establishing the Participant GitHub Repository

A GitHub repository, GW-SMHS-BIOC6223 was initialized with a README (<https://github.com/biocompute-objects/GW-SMHS-BIOC6223/blob/master/README.md>) documenting the repository’s purpose and protocol for creating a project board, resolving issues, and addressing blockers. This repository was established with the intention to add an additional tool to their skillset, start their online portfolios, facilitate BCO feedback, and to train participants to utilize GitHub as a means of issue resolution in a collaborative environment.

To provide participants the opportunity to expand their knowledge and learn an additional tool, the free code-sharing and publishing service, GitHub was utilized to facilitate support and feedback on evaluation deliverables (BCOs). Robust help documentation (<https://github.com/about>) was available on GitHub for participant reference. As the participants uploaded their BCOs, they received direct and actionable feedback in the form of issues (<https://github.com/biocompute-objects/GW-SMHS-BIOC6223/issues>). This was done to make participants comfortable working in a GitHub environment.

GitHub was selected for training module management (publicly available documentation of the evaluation, weekly deliverables, high level diagrams, etc. and a location for participants to upload their work for direct feedback) and easy transfer of feedback to the appropriate BioCompute repository (for example, issues could be reviewed and sent to the BCO Editor Repository in GitHub (<https://github.com/biocompute-objects/bco_editor>) with descriptive labels like “feedback” and “BCO Editor”.

The GW-SMHS-BIOC6223 repository contains information such as

1) Information and criteria for sourcing publications for BCO creation
(<https://github.com/biocompute-objects/GW-SMHS-BIOC6223/blob/master/docs/1_SelectPublication.md>)

2) Organizing pipeline information
(<https://github.com/biocompute-objects/GW-SMHS-BIOC6223/blob/master/docs/2_GroupDiscussion.md>)

3) Instructions to draft BCOs in the BCO Editor
(<https://github.com/biocompute-objects/GW-SMHS-BIOC6223/blob/master/docs/3_DraftBCO.md>)

4) Researching reproducibility of the pipelines in platforms such as HIVE and CGC (<https://github.com/biocompute-objects/GW-SMHS-BIOC6223/blob/master/docs/4_FurtherResearch.md>)

5) How to implement reviewer feedback via GitHub and the BCO Editor
(<https://github.com/biocompute-objects/GW-SMHS-BIOC6223/blob/master/docs/5_ModifyBCO.md>)

#### Starting participants on GitHub: Team, Project Board, Issue resolution

Once the participants had created a GitHub account, they were added to the “Participants-[F19]” team in the BioCompute Repository. Participants first created a project in the Projects tab of the GW-SMHS-BIOC6223 repository, and the Project board name was established according to the template: "[fullname]" (Example: JanishaPatel), along with a project description up to their discretion. To bolster their projects with basic project management structure, they selected the "Basic Kanban" project template (integrated into GitHub); this template established columns for “to do”, “in progress” and “done”. The project board enabled them to sort tasks, plan projects, automate workflows, track progress, share status, and retire work once complete. For more documentation on project boards, participants were directed to GitHub documentation for help.

User BCOs were saved and downloaded from the BCO Editor, then were uploaded to GitHub GW-SMHS-BIOC6223 repository. The BioCompute Technical reviewer provided feedback in the form of GitHub issues, which BCO creators had access to on their Project Boards. Users resolved these issues and submitted their corrected and reviewed BCO to the pFDA challenge. Participants were able to reply to the feedback for clarification or further discussion up until 12hours before the pFDA challenge deadline.

#### Blockers/Questions

Throughout this process, users were asked to create GitHub issues ([https://github.com/biocompute-objects/GW-SMHS-BIOC6223/issues](https://github.com/biocompute-objects/GW-SMHS-BIOC6223/issues/new)) when they encountered blockers, had questions, or provided feedback. For additional BioCompute help, participants were directed to the BioCompute Specification repository (<https://github.com/biocompute-objects/BCO_Specification>). For additional GitHub help, participants were directed to GitHub documentation ([https://guides.github.com/activities/hello-world/)](https://guides.github.com/activities/hello-world/).

1. User Feedback Survey Results

User feedback provided clear insights for improving the training module. Training module survey responses can be seen in Figure 4. Feedback included sections of the training module users found most useful or valuable: performing and organizing a research project via computational means, navigating different types of scientific databases and resources for exploration of scientific knowledge. When asked how they would improve the training module, users responded that they’d prefer to extend the training module and have more in-person training on different bioinformatics tools.

# Abbreviations

NGS: Next-Generation Sequencing

BCO: BioCompute Object

HTS: High-Throughput Sequencing

HIVE: High-Performance Integrated Virtual Environment

CGC: Cancer Genomics Cloud

JSON: JavaScript Object Notation

USFDA: US Food and Drug Administration

pFDA: precisionFDA

GUI: Graphical User Interface

SB: Seven Bridges

SRA: Sequence Read Archive

CWL: Common Workflow Language

EPA: Environmental Protection Agency

STRIDES: Science and Technology Research Infrastructure for Discovery, Experimentation, and Sustainability
